# p65BTK Is a Novel Biomarker and Therapeutic Target in Solid Tumors

**DOI:** 10.3389/fcell.2021.690365

**Published:** 2021-06-07

**Authors:** Emanuela Grassilli, Maria Grazia Cerrito, Sara Bonomo, Roberto Giovannoni, Donatella Conconi, Marialuisa Lavitrano

**Affiliations:** Laboratory of Molecular Medicine, Department of Medicine and Surgery, University of Milano-Bicocca, Monza, Italy

**Keywords:** p65BTK, targeted therapy, drug resistance, colon cancer, lung cancer, ovarian cancer

## Abstract

Bruton’s tyrosine kinase (BTK) is a non-receptor intracellular kinase playing a key role in the proliferation and survival of normal and malignant B-lymphocytes. Its targeting by Ibrutinib, the first specific inhibitor, represented a turning point for the therapy of certain types of B-cell leukemias/lymphomas and several more BTK inhibitors are today in the clinic or advanced clinical trials. BTK expression was successively found to occur also outside of the hematopoietic compartment. In fact, we identified p65BTK, a novel 65 kDa isoform lacking an N-term stretch of 86 amino acids (compared to the 77 kDa protein expressed in B cells) as highly expressed in colon cancer patients. We demonstrated that p65BTK is a powerful oncogene acting downstream of the RAS/MAPK pathway and necessary for RAS-mediated transformation. Notably, the kinase domain is conserved and therefore inhibited by the available BTK-targeting drugs (Ibrutinib, Spebrutinib, etc.) which we used to demonstrate that p65BTK is an actionable target in drug-resistant colorectal carcinomas. We found p65BTK expressed also in >50% non-small cell lung cancers (NSCLC) and demonstrated that it is an actionable target in *KRAS*-mutated/*EGFR*-wild type drug-resistant NSCLC models (for which no targeted therapy is available). We also reported a significant correlation between p65BTK expression and low-grade tumors and overall survival of patients with grade III gliomas and showed that its targeting induced a significant decrease in the viability of in glioma stem cells. Finally, in ovarian cancer patients, p65BTK expression levels correlate with early relapse and shorter progression-free survival, both indicators of resistance to therapy. Remarkably, Ibrutinib is more effective than standard of care (SOC) therapeutics in *in vitro* and *ex vivo* settings. On the whole, our preclinical data indicate that, depending on the tumor type, BTK inhibitors used alone can induce cytotoxicity (gliomas), be more effective than SOC chemotherapy (ovarian cancer) or can kill drug-resistant tumor cells when used in combination with SOC chemotherapy (colon cancer and NSCLC) or targeted therapy (NSCLC and ovarian cancer), thus suggesting that p65BTK may be an actionable target in different solid tumors. In addition, our data also give the proof-of-concept for starting clinical trials using BTK inhibitors, alone or in combination, to improve the therapeutic options for solid tumors treatment.

## Introduction

Bruton’s tyrosine kinase (BTK) is a 77 kDa non-receptor kinase originally identified as being defective in the primary immunodeficiency X-Linked Agammaglobulinemia (XLA. OMIM #300755) (Tsukada et al., 1993). Loss-of-function mutations in the *BTK* gene were shown to impair the ability of B-cell precursors to differentiate, with a consequent severe decrease of mature B lymphocytes, both in the bloodstream and in the lymph node sites. Accordingly, XLA patients showed a complete absence of circulating B cells and no antibody production, rendering them particularly prone to bacterial, but not viral, infections. BTK function was therefore recognized as essential for B cell development and functionality because of its action downstream of the B-cell antigen receptor (BCR). Its expression was successively found in all the cell lineages of the hematopoietic compartment, except for T cells and for long time it was generally believed that its expression and function were limited to bone marrow-derived cells (Mohamed et al., 2009). In the last decade or so, the finding that BTK is overexpressed/hyperactive in some B-cell malignancies led to the rapid development and approval of Ibrutinib (Imbruvica), the first BTK inhibitor, for treating patients with relapsed/refractory chronic lymphocytic leukemia and mantle cell lymphoma (MCL) (Cameron and Sanford, 2014). Single-agent therapy with Ibrutinib is generally well tolerated and effective, but resistance usually develops (Zhang et al., 2015), which led to the testing of combination therapies and the development of other inhibitors, recently approved or still in clinical trials (Byrd et al., 2016; Bond and Woyach, 2019; Sawalha et al., 2020; Sharman et al., 2020). The race to the development of a number of diverse inhibitors has also been fueled by evidence pointing to the efficacy of BTK inhibition in other B cell malignancies and in auto-immune diseases (Lorenzo-Vizcaya et al., 2020; Dolgin, 2021; Zarrin et al., 2021). In addition, inhibiting BTK affects more than BCR-driven signaling: in fact, BTK also plays a critical role in the downstream signaling pathways for the Fcγ receptor in monocytes (Ren et al., 2016), the Fcε receptor in granulocytes (Kneidinger et al., 2008; Krupa et al., 2013, 2014), the TLR in myeloid cells and in myeloid-derived suppressor cells (Rip et al., 2019), all of them crucial components of the tumor microenvironment. These findings, together with Ibrutinib being also an EGFR family inhibitor (Gao et al., 2014; Grabinski and Ewald, 2014; Wu et al., 2015; Chen et al., 2016; Wang et al., 2016; Rauf et al., 2018), paved the way for clinical trials of BTK inhibitors in solid tumors (NCT03525925; NCT02403271; NCT02899078; NCT03646461; NCT03332498). However, recent data indicated that BTK inhibitors might be helpful in solid tumors also because of a direct effect: in fact, BTK or two different isoforms of the kinase, BTK-C and p65BTK, have been discovered as highly expressed in several solid tumors.

BTK-C has been identified by performing a kinase-directed shRNA-based screen aimed at the identification of novel survival factors in breast cancer cells. The transcript identified in breast cancer cells revealed to be identical to an automated computationally predicted (GNOMON) sequence named BTK-cra-C; hence the authors referred to this isoform as BTK-C. The authors demonstrated that ablation of its function either by siRNA-mediated downregulation or pharmacological inhibitors induced apoptosis of breast cancer cells whereas its overexpression afforded protection from apoptosis induced by Doxorubicin (Eifert et al., 2013). The same group successively confirmed a survival role for BTK-C also in prostate cancer cells where its downregulation by RNAi or inhibition with BTK inhibitors induced apoptosis via induction of several apoptosis-related gene, as assessed by microarray analysis. Instead, its overexpression was associated with induction of genes with functions related to cell adhesion, cytoskeletal structure and the extracellular matrix implying a role for BTK signaling in cell adhesion and migration (Kokabee et al., 2015). A role in migration of prostate cancer cell has indeed been confirmed by Zhu et al. (2020) who reported that Ibrutinib significantly inhibited cell proliferation, migration and invasion of prostate cancer cells as well as protein synthesis of MMP-2 and MMP-9. In addition, strong expression of BTK was detected in prostate cancer tissues, especially in tumors from prostate cancer patients with bone metastasis.

Li et al. (2018) using a proteomics approach, identified the known 77 kDa BTK isoform as an ALK interaction partner able to potentiate ALK-mediated signaling in neuroblastoma. They showed that Ibrutinib treatment can effectively inhibit the growth of neuroblastoma xenograft in nude mice, and the combination of ibrutinib and the ALK inhibitor crizotinib further enhanced the inhibition. In addition, they reported that high expression of BTK correlates with poor relapse-free survival probability of neuroblastoma patients. Pikatan and colleagues found high percentages of cells with strong BTK expression in undifferentiated (57.1%) and poorly differentiated neuroblastoma (88.7%) and suggested BTK as a potential biomarker of neuroblastoma differentiation status. Moreover, the analyses of two different microarray datasets confirmed BTK overexpression in neuroblastoma patients which significantly correlated with a worse 3-year overall survival (OS) (Pikatan et al., 2020). To note, high BTK expression occurred preferentially in MYCN-amplified neuroblastoma cases. In addition, they showed that pharmacologic or genetic inhibition of BTK reduced neuroblastoma cells survival and their migratory and invasive abilities *in vitro* and reduced tumor growth *in vivo*. In this study it was assumed that the BTK expressed in neuroblastoma was the 77 kDa isoform. To identify drug sensitivity effects associated with specific mutational backgrounds, Chong and colleagues integrated the genomic profile of 17 esophageal tumor-derived cell lines with drug sensitivity data from small molecule inhibitor profiling. Putative targets were then verified by interrogating recently described RNA interference screen data for the same tumor cell lines. By this approach they identified and validated a vulnerability in the inhibition of BTK in MYC-amplified esophageal tumor cell lines (Chong et al., 2018). Also, in this type of tumor the isoform that could be exploited as a therapeutic target was the 77 kDa one. Very recently Pan et al. reported that BTK expression levels are associated with a higher risk of bladder cancer progression and are higher in cancer stem cell-like spheroids compared to bladder cancer cell cultures. They also pinpointed a role for BTK in cancer cell migration and resistance to chemotherapy by using genetic and chemical ablation of BTK function (Pan et al., 2020). On the whole, data reported so far about the role of BTK in solid tumors suggest that its increased expression is associated to worse prognosis, to advanced cancer metastatic potential, drug-resistance and stemness.

Our lab discovered the p65BTK isoform and in this review, we will discuss its role in solid tumors, with a particular emphasis of the importance of inhibiting it as a novel important anti-cancer therapy.

## The Discovery of p65BTK

Drug resistance is the main reason of the failure of the current anti-cancer therapies which ultimately leads to the death of cancer patients. One of the most relevant causes of drug resistance is the loss of p53 activity, which abolishes the apoptotic response to many anticancer agents (Kastenhuber and Lowe, 2017). To identify specific kinases whose loss is synthetic lethal with chemotherapy we performed a kinase-directed shRNA screen in colorectal cancer (CRC) cells resistant to 5-Fluorouracil (5-FU) due to the loss of p53 (Grassilli et al., 2013) and, among the strongest hits, we identified a novel BTK isoform, which we dubbed p65BTK from his apparent molecular weight (Grassilli et al., 2016). p65BTK protein was found to be abundantly expressed in CRC tissues and CRC cell lines. Its encoding mRNA contains a 1st exon that is different from the one present in the messenger encoding the 77 kDa protein expressed in the hematopoietic compartment, now indicated as isoform 1. BLAST alignment showed that the 300 bp-long exon mapped 15,192 bp upstream of the known *BTK* 1st exon and is very close (225 bp) to the start of *RPL36A* gene, transcribed in the opposite sense. According to the Genome Data Viewer database of the NCBI a CpG island 200 bp upstream from the start of the 1st exon indicates an independent promoter, other than the one used to start the transcription of p77BTK. Notably, the same mRNA has been independently identified by Conklin’s group through a similar approach i.e., a kinase-directed shRNA-based screen aimed in their case at the identification of novel survival factors in breast cancer cells (Eifert et al., 2013). However, the protein expressed from the mRNA in breast cancer cells is 80 kDa (BTK-C), due to the utilization of an ATG located very proximal to the start of the exon 1. In CRC cells the ATG used to start the translation is not the one in the first alternative exon, nor the one used to translate p77 (located in the 2nd exon and thus present in the p65BTK-encoding mRNA) but instead an in-frame one located in the 4th exon. It has to be noted that, in *in vitro* expression system, within the context of p77BTK mRNA, the ATG in the 4th exon can also be recognized as a starting codon, albeit with much lower efficiency (Grassilli et al., 2016).

As a result of translation starting from the 4th exon, p65BTK lacks the first 86 amino acids of the N-term, i.e., most of the PH domain where phosphatidylinositol-3,4,5-trisphosphate bind (allowing BTK translocation to the membrane and its activation) and several other proteins have been reported to interact with, most of them negative regulators. Therefore, it is expected that p65BTK would be regulated/activated differently than p77BTK and that it would be abundantly expressed and activated. In fact, Andreotti’s lab demonstrated by structural studies that the lack of the N-terminal leads to increased levels of spontaneous p65BTK activation (Joseph et al., 2017). Moreover, given the absence of the most important sites of regulation and interaction located in the N-terminal part of the 77 kDa isoform, it might be anticipated that p65BTK is likely to be involved in different signaling pathways. In addition, the absence of the first N-terminal 86 amino acids might lead to different conformational structure(s) and/or post-translational modification crucial for protein-protein interaction. Therefore, only a proteomic approach to identify interactors of p65BTK will shed light on its substrates and in which signaling pathway p65BTK is involved.

**FIGURE 1:**
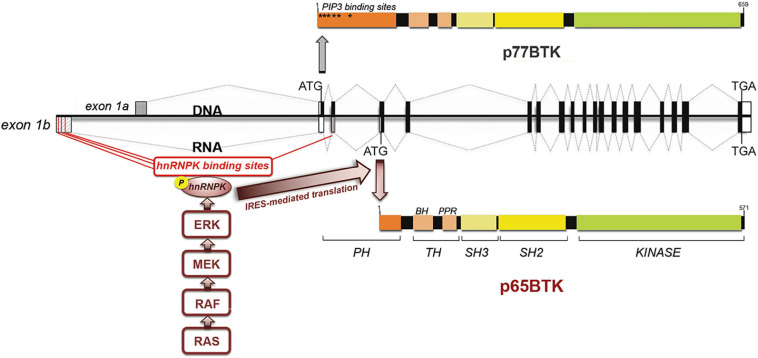
p65BTK organization and regulation of expression. BTK gene and mRNAs encoding p77BTK and p65BTK. ATG: start codons, Black/white boxes: translated/untranslated exons. Red lines: hnRNPK-binding sites. The translation of the p65BTK-encoding mRNA occurs via an IRES-mediated mechanism and requires the binding of phospho-hnRNPK to the 5′UTR. The phosphorylation of hnRNPK in turns depends on ERKs, activated downstream of the RAS/MAPK pathway.

p65BTK-encoding mRNA is expressed at very low levels and is characterized by a 336bp-long 5′UTR crucial for regulating p65BTK translation and protein abundance and contain four putative hnRNPK binding sites. hnRNPK is a RNA-binding nuclear protein involved in chromatin remodeling, transcription, splicing, translation, and mRNA stability (Bomsztyk et al., 2004) that is overexpressed and aberrantly localized in the cytoplasm in CRC (Carpenter et al., 2006). Analysis of the 5′UTR by an RNA structure prediction software revealed a complex folding pattern, with the other ATGs used for translation of BTK-C and BTK hidden in hairpin loops, leading us to hypothesize that 5′UTR-bound hnRNPK would promote a 3D-structure favoring the ribosome to start the translation from the ATG located in the 4th exon. Indeed, p65BTK-encoding mRNA binds hnRNPK, and in particular a phosphorylated form, dependent on the activation of the MAPK/ERK pathway. Accordingly, p65BTK expression levels were decreased by MEK inhibitors via diminution of phospho-hnRNPK. Proteins can be translated by both cap-dependent and internal ribosome entry site (IRES)-dependent mechanisms. The latter is switched on to maintain the expression of specific proteins during pathological situations when cap-dependent translation is compromised, such as following heat shock or during mitosis, hypoxia, differentiation and apoptosis (Spriggs et al., 2008). Translation of p65BTK was found to be IRES-dependent, being phospho-hnRNPK binding to the IRES sequence necessary for translation to occur. In addition, expression levels of p65BTK are post-transcriptionally regulated since very low levels of p65BTK-encoding mRNA were detectable in patients’ colonic tissue, both tumoral and normal, whereas the protein was highly expressed only in cancer and not in normal tissue. Thus, expression levels of p65BTK are tightly regulated at the translational level by two intertwining mechanisms (Figure 1), both of which are dysregulated in many cancer cells. In fact, MAPK/ERK pathway is usually dysregulated as a consequence of RAS mutation, occurring in up to 30% of CRCs (Prior et al., 2020). Considering all types of cancers, hyperactive MAPK signaling occurs in over 85% of cancers, which is caused directly by genetic alterations of its upstream activators or components, including RTKs, RAS, and BRAF, or indirectly by other regulators of the pathway (Yuan et al., 2020).

Given the abundant expression of p65BTK in CRC tissues and that most oncogenes are translated via an IRES-dependent mechanism (Spriggs et al., 2008; Gao and Roux, 2015) p65BTK appeared to be a putative oncogene. In fact, when testing the transforming activity of p65BTK it was unexpectedly found that this novel isoform is a stronger oncogene than RAS and is an obligate effector of RAS-mediated transformation itself. In addition, p65BTK transforming activity resulted dependent on active ERK1/2 and, accordingly, p65BTK overexpression in CRC tissues was found to correlate with ERK1/2 activation. Performing the transformation assays it was observed that overexpression of active RAS induced the expression of endogenous p65BTK, as expected as a consequence of MAPK/ERK hyperactivation. Surprisingly, also overexpression of the coding sequence of p65BTK (thus independent from RAS/ERK-mediated regulation) induced endogenous RAS and the use of a RAS inhibitor, as well a dominant negative form of RAS, blocked p65BTK-mediated transformation (Grassilli et al., 2016). These results point to the existence of a positive feed-back mechanism. We have not explored such mechanism but it could be hypothesized that it might occur via transcriptional regulation, since the murine homologue of ARID3A (Bright) has been demonstrated to critically require interaction with functional BTK in order to enhance immunoglobulin transcription (Rajaiya et al., 2005).

Altogether these findings pointed out p65BTK as a novel oncogene downstream of RAS and a putative actionable target in mutant (mt) *RAS* tumors, for which no targeted therapy is yet available. In fact, despite that the first small molecules directly targeting a specific RAS mutant (G12C) are currently undergoing clinical evaluation several different strategies are still being pursued, such as strategies directly disrupting RAS interaction with activating GEFs, targeting the scaffolding phosphatase SHP2 and targeting of downstream effectors of RAS (Stalnecker and Der, 2020). Along this line targeting p65BTK has several advantages: for example, the fact that the kinase domain is conserved and thus amenable to inhibition by the available BTK-targeting drugs already in the clinics (Ibrutinib, Zanubrutinib, Acalabrutinib) and the many more actually in advanced phase of clinical trial (Spebrutinib, Tolebrutinib, Evobrutinib, etc.). Notably, BTK inhibitors have been in the clinic for a few years and their clinical tolerability is already known, thus indicating a potential for rapidly re-positioning of BTK inhibitors for the therapy of solid tumors.

Remarkably, p65BTK would be the ideal target. In fact, it is known that XLA patients bears mutations mostly in the kinase domain of BTK (Smith, 2017), that is conserved in p65BTK. Therefore, it can be assumed that these patients do not express also a functional p65BTK. Apart from the inability to produce immunoglobulins in response to bacterial infections the lack of functional BTK does not have other remarkable side effects, suggesting that targeting p65BTK is not harmful for normal tissues. At variance targeting p65BTK in tumoral cells appears to be lethal. In fact, we have not been able in more than a decade of experiments to obtain stable cell lines not expressing p65BTK: even though at 48hs after transfection with a selectable shBTK plasmid we could observe a significant reduction of the protein, at the end of the selection period the surviving cells were the ones re-expressing p65BTK, indicating that cells developed compensatory systems to avoid the silencing of the protein. Similarly, in experiments using siRNAs, where the silencing is transient, we have never been able to suppress p65BTK expression completely. Finally, we also tried to establish cell lines expressing a kinase-dead dominant negative version of p65BTK but in this case all cells were dead by 48hs post-transfection, even before starting the selection (unpublished data), further reinforcing the notion that complete loss of p65BTK is not compatible with tumor cell survival. Accordingly, we observed that Ibrutinib treatment decreased the levels of anti-apoptotic Mcl-1 and at the same time strongly increased its cleaved form. In addition, p65BTK inhibition increased the levels of two pro-apoptotic proteins: PUMA and the extra-long isoform of BIM (Lavitrano et al., 2020). p65BTK seems therefore to be an upstream regulator of pro-survival/anti-apoptotic pathways.

## p65BTK Targeting in Colon Cancer

Colorectal cancer is the fourth leading cause of death from cancer worldwide and, despite significant progress in early detection and novel combination treatments added to classic chemotherapy, a high proportion of patients rapidly become drug-resistant and eventually succumb to metastatic disease (Hammond et al., 2016). Therefore, identifying novel biomarkers and actionable targets is a priority to further improve the therapeutic response and survival of CRC’s patients. To assess the actionability of p65BTK in CRC we performed several studies on different cohorts of patients for a total of 353 patients. First, by IHC high levels of expression at the protein level were shown to correlate with activated ERKs in a small number of specimens (*n* = 12) of CRC tissues (Grassilli et al., 2016). p65BTK expression was also quantified in three different cohorts of CRC patients: samples from two cohorts of 19 and 61 CRC patients were analyzed by ELISA and those from a third cohort of 174 CRC patients were analyzed by IHC (Lavitrano et al., 2020). Finally, p65BTK expression was evaluated by IHC on a cohort of 87 stage III CRC patients (Basile et al., 2019). ELISA analysis of normal/tumor sample pairs showed that p65BTK expression levels significantly increased with histological tumor grade. IHC analysis demonstrated p65BTK expression in adenoma and adenocarcinoma but not adenosquamous carcinoma. Moreover, considering both, the staining intensity (absent, weak, moderate or strongly positive) and the percentage of stained tumor cells in the sample, a medium-to-strong intensity was found in a percentage of patients’ samples ranging from 65.5 to 81.8% in stage I–III (Lavitrano et al., 2020). In particular, stage III patients expressing the highest intensity of p65BTK in >80% cells had the worst prognosis in terms of disease-free survival (DSF) and OS, pointing out p65BTK as a potential prognostic marker in stage III CRC (Basile et al., 2019).

Given that we identified p65BTK in synthetic lethal loss-of-function screen aimed at identifying actionable targets in drug-resistant cells, the role of the novel isoform was experimentally explored in *in vitro* (cell lines), *ex vivo* (patient-derived organoids) and *in vivo* (tumor xenografts) models of 5FU-resistant CRCs using both, functional genetic approaches (shRNA/siRNA) and chemical approaches (BTK inhibitors). In all models p65BTK targeting restored the apoptotic response to 5-FU of drug-resistant cells and *in vivo* administration of Ibrutinib in combination with 5-FU significantly reduced tumor growth (Lavitrano et al., 2020). Mechanistically, we demonstrated that blocking p65BTK in drug-resistant cells abolished a 5-FU-elicited TGFB1 protective response and triggered E2F-dependent apoptosis, thereby identifying p65BTK as an important player in the TGFB and E2F1 pathways. It has been repeatedly reported that Ibrutinib inhibits also other kinases, among them EGFR, presenting the conserved Cys481 in the kinase domain, that is the binding site for the molecule (Gao et al., 2014; Grabinski and Ewald, 2014; Chen et al., 2016). However, the possibility that the effect of Ibrutinib might be via inhibition of the EGFR rather than by blocking p65BTK was clearly ruled out (Lavitrano et al., 2020). In fact, CRC cell lines with mutation in the RAS/MAPK pathway, intrinsically resistant to EGFR inhibitors, were used. Accordingly, EGFR inhibitors, such as Afatinib and Poziotinib, did not induce significant cytotoxicity either singularly or combined with 5-FU, whereas apoptosis was instead elicited when EGFR-inhibiting doses of Ibrutinib were added to 5-FU; moreover, adding EGFR inhibitors to Ibrutinib induced significant cytotoxicity in EGFR inhibitors-resistant CRC cell lines. Significantly, re-sensitization to 5-FU occurred following reduction of p65BTK levels by functional genetic approaches and conversely, protection from 5-FU-induced cytotoxicity of sensitive cells was afforded by overexpression of p65BTK, but not its kinase-dead version. Finally, BTK inhibitors with different mechanisms of action were used. Notably, Ibrutinib, Spebrutinib (formerly AVL-292) and ONO-4059 (now known as Tirabrutinib) bind covalently to the Cys481 in the ATP-binding domain, CGI-1746 binds to unphosphorylated BTK in the SH3 domain, thus stabilizing the protein in an inactive conformation and preventing its auto-phosphorylation, and RN486 interacts with K430 a residue critical for kinase activity. Remarkably, independently of the mechanism of action, any inhibitor in combination with 5-FU had a strong synergistic and cytotoxic effect. It has to be noted that to kill CRC cell lines the inhibitors were used at concentrations higher than those reported in the literature for B-cells leukemias and lymphomas. This might be explained by many reasons, among which different drug pharmacokinetics. For example, Ibrutinib is known to be metabolized by CYP3A4 and 3A5 (Scheers et al., 2015) both overexpressed in a large number of CRCs but not in B-cell neoplasia (Lolodi et al., 2017). In addition, inter-individual variability of CYP3A activity linked to different genetic polymorphisms has also been shown (Hohmann et al., 2016). In line with this, it has been reported that in patients with high CYP3A activity ibrutinib dosage must be increased to be therapeutically effective (Finnes et al., 2017). Notably, the results obtained using patient-derived organoids, which are considered a good surrogate for precision medicine based on patient stratification, confirm an inter-individual variability in the response to Ibrutinib and Spebrutinib. This might be due to individual differences in drug pharmacokinetics or to the fact that organoids capture the heterogeneity of the real patients’ cancer cell population and mimic the 3D *in vivo* situation. Careful studies should therefore be performed to understand the reason(s) of these differences, especially in the view of starting clinical trials with BTK inhibitors for treating solid tumors, being the caveat that one inhibitor might not fit-it-all.

## p65BTK Targeting in Lung Cancer

Despite recent introduction of targeted therapies and immune-checkpoint inhibitors combined with chemotherapy lung cancer still accounts for about 1/3 of all cancer-related deaths worldwide, thus being the number one killer cancer (Wong et al., 2017). Several actionable cancer genes such as *EGFR*, *ALK, ROS1, BRAF* have been identified, for which specific targeted therapies exist; however, >40% of patients’ tumors do not have alterations in actionable genes thus excluding for these patients the possibility of being treated with a targeted therapy (Chan and Hughes, 2015). In addition, even though about 1/3 of lung cancers possess a mutation of *KRAS*, no specific inhibitor is available in the clinic so far; moreover, mutations in *KRAS* are mutually exclusive with *EGFR* mutations and are associated with resistance to chemotherapy or EGFR inhibitors (Eberhard et al., 2005), thus leaving a significant percentage of lung cancer patients with a potentially actionable mutation with few therapeutic options. Interestingly, in a cohort of 383 chemo- and/or radio-naïve non-small cell lung cancer (NSCLC) patients we found that p65BTK was expressed in more than half of them and its levels were significantly higher in never-smokers and in *EGFR*-wt tumors, therefore suggesting that p65BTK could be a novel target in advanced NSCLC E*GFR*-wt non-smokers patients that are not eligible for targeted therapy (Giordano et al., 2019). Moreover, in NSCLC cell lines and tumors from a mouse model of *Kras*-driven lung cancer, p65BTK levels were highest in samples with a mutation in *KRAS* or in the RAS/MAPK pathway and – accordingly to what previously shown in CRC – p65BTK expression was dependent on the RAS/MAPK pathway, indicating that p65BTK might be an actionable target in a significant number of lung tumors. In fact, given that we showed that BTK inhibitors significantly hampered cell proliferation and clonogenicity of all the cells lines with hyper-activation of the MAPK pathway due to different genetic defects (double mutation L858R/T790M in the *EGFR*; *ALK* translocation; *KRAS* mutation) p65BTK actionability may encompass even a broader spectrum of patients than those simply harboring a *KRAS* mutation. Finally, we showed that, independently of the *EGFR/KRAS* mutational status, BTK inhibitors are strongly synergic with both EGFR inhibitors and SOC chemotherapy by turning a mild anti-proliferative effect in a cytotoxic one. On the whole, if our preclinical data will be confirmed by clinical trial we might envisage that a significant numbers of NSCLC patients previously ineligible for targeted therapy might be offered a novel therapeutic approach based on p65BTK inhibition.

## p65BTK Targeting in Glioblastoma

Gliomas are the most common central nervous system tumors and include astrocytoma, ependymoma, glioblastoma, oligodendroglioma and various subtypes/combinations. Glioblastoma (GBM) is a grade IV astrocytoma that represents about 50% of all brain tumors and is the most frequent glioma. Most GBM arise *de novo*, whereas secondary GBM tumors commonly develop from lower grade gliomas; independently from the origin, GBM has the worst prognosis (survival at 5 years <5%) mainly due to the fact that GBM is chemo- and radio-resistant (Omuro and DeAngelis, 2013). From a cellular and molecular point of view, GBMs present high heterogeneity which contributes to recurrence and therapeutic resistance (Patel et al., 2014; Reimunde et al., 2021). Three main molecular alterations can be recognized in GBMs: the dysregulation of growth factor signaling (in 57% of GBMs gain-of-function mutations and/or focal amplification of EGFR occur with an increase in the aggressiveness of these gliomas), the activation of the PI3K pathway and the inactivation of the p53 or RB (Verhaak et al., 2010). In particular, early p53 mutation is a distinctive sign of grade II gemistocytic astrocytoma, a frequent precursor of GBM associated with an aggressive behavior, a rapid progression and reduced OS compared to other cytotypes. In addition, IDH mutation occurs early during the development of the tumor and is the main biomarker to identify and classify GBM (Louis et al., 2016).

Analyzing a large cohort of 71 patients with glial tumors our group found p65BTK expression in 1/5 of cases; notably BTK expression significantly correlates with low-grade tumors (*p* ≤ 0.05) and OS of patients with grade III gliomas (*p* ≤ 0.05), suggestive of worst prognosis. Interestingly, co-expression of EGFR and p53 (indicative of mutated p53) was present in 92.3% of BTK-positive patients. Remarkably, p65BTK was expressed exclusively in gemistocytic cells and this specificity does not depend on grade and histotypes. The expression of p65BTK was present in all gemistocytic cells in all the examined samples, suggesting that p65BTK might be a specific biomarker of a particularly aggressive subset of GBM, characterized by unfavorable prognosis (Jung et al., 2011). Accordingly, in an orthotopic mouse model of human GBM – obtained injecting glioma stem cells (GSC) in immunodeficient mice – p65BTK expression remained restricted exclusively to gemistocytic cells. Finally, *in vitro* experiments performed on GSC lines, isolated from GBM showed that Ibrutinib significantly reduced metabolic activity and mitotic index and increased cell death (Sala et al., 2019), thus indicating that p65BTK might be an actionable target in GBM.

A previous study reported that BTK was overexpressed in glioma and Ibrutinib suppressed glioma cell growth *in vitro* and *in vivo* (Yue et al., 2017). However, the authors did not investigate which isoform was expressed in the 11 glioma samples studied. In addition, they investigate the expression profile of BTK in human normal brain and glioma samples using GEO microarray dataset (GSE16011) which do not distinguish between the two isoforms, given that the probe set used for BTK spans all over the mRNA length. The fact that high BTK expression levels were associated with poor prognosis in patients with lower grade glioma is in agreement with our data and would suggest that the isoform expressed in glioma is indeed p65BTK.

## p65BTK Targeting in Ovarian Cancer

Ovarian cancer is the most lethal gynaecological malignancy in western countries: in fact, at the moment no early distinctive symptoms and specific screening tests exist and, as a consequence, in most cases the diagnosis is made at a very late stage (Sato and Itamochi, 2014). In addition, despite the emergence of a novel biomarker such as *BRCA1/2* mutation that has made possible the use of PARP inhibitors for therapy, this option is still limited in the clinical practice given that less than 15% of ovarian cancers carries such mutation (Cortez et al., 2018). Beside *BRCA1/2* mutation no other actionable targets have been reported so far and SOC treatment consists of surgery followed by platinum/taxane chemotherapy, with the possible addition of anti-angiogenic therapy (Bevacizumab). Overall, the risk of recurrence ranges from 60% to 85% and drug resistance emergency is very high. Analyzing a cohort of ovarian cancer patients we found that high p65BTK expression in patient’s samples correlates with early relapse and a worst PSF, both indicators of resistance to therapy; these data suggest that p65BTK might be a prognostic biomarker. In addition, we also showed that it might be an actionable target: in fact, using *in vitro* (cell lines) and *ex vivo* (cells freshly dissociated from patient-derived xenografts and patient-derived cancer) experimental systems we found that BTK inhibitors strongly affected proliferation and survival of ovarian carcinoma cells (Conconi et al., 2018). Notably, the treatment of patient-derived cancer cells with Ibrutinib was more effective in decreasing cell survival than SOC treatments (carboplatin, paclitaxel, bevacizumab) suggesting that p65BTK targeting might be a useful therapeutic approach for tumors scarcely responsive to SOC therapy. Further studies are in progress to understand whether, as already shown in CRC and NSCLC models, p65BTK expression levels correlate with RAS/MAPK dysregulation and whether BTK inhibitors are synergic with other targeted therapy, such as EGFR inhibitors, or SOC therapy.

## Conclusion/Perspectives

Since the discovery that targeting BTK leads to remarkable therapeutic responses in several B cell malignancies BTK inhibitors are increasingly replacing chemotherapy-based regimen, especially in patients with CLL and MCL (Burger, 2019). Given that BTK activity is essential in driving B cells proliferation it plays a pivotal role also in autoimmune diseases. For this reason the field of BTK inhibitors has developed rapidly in the last 10 years, and some of them are already in the clinics whereas others are in advanced phase of clinical trial. Recently, it has been reported from different laboratories that BTK and its new isoforms are expressed and actionable targets in solid cancers, indicating a potential for re-positioning BTK inhibitors for the therapy of solid tumors.

In particular, we discovered the novel p65BTK isoform and demonstrated that its expression depends on the RAS/MAPK pathway. Accordingly, we showed that its targeting in mt-*KRAS* colon and lung cancer cells impaired their proliferative and clonogenic ability. Notably, no therapy targeting mt-*KRAS* is yet available, even though the first small molecule specifically targeting *KRAS^*G*12*C*^* mutant is currently being tested in clinical trials (Stalnecker and Der, 2020). In addition, we showed the advantage of using BTK inhibitors in several tumor models over-expressing p65BTK and resistant to target therapy or SOC chemotherapy. In fact, depending on the type of cancer, BTK inhibitors used alone resulted more effective than SOC chemotherapy in reducing cell proliferation (ovarian cancer) or restored sensitivity to SOC chemotherapy (colon cancer and NSCLC) and/or targeted therapy (NSCLC) of drug-resistant tumor cells. Of particular interest the finding that inhibiting p65BTK could overcome the resistance to drugs targeting upstream nodes in the RAS/MAPK pathway. CRCs bearing a *BRAF^*V*600*E*^* mutation are intrinsically resistant to BRAF inhibitors due to feedback activation of EGFR (Prahallad et al., 2012) therefore trials are undergoing using EGFR and MEK inhibitors (Ros et al., 2021). Previous experience in melanomas, where double targeting with BRAF and MEK inhibitors is already in the clinic, showed that eventually resistance occurs due to compensatory MAPK pathway re-activation (Kakadia et al., 2018). Based on our findings we speculate that the inhibition of a downstream effector, such as p65BTK, possibly in combination with BRAF and/or MEK inhibitors, would prevent and/or circumvent the onset of drug resistance. *Ex vivo* and *in vivo* studies are undergoing to test this hypothesis.

In conclusion, the data obtained in our laboratory provides proof-of-concept that targeting p65BTK is therapeutically advantageous in several solid tumors and indicate that starting clinical trials using BTK inhibitors, alone or in combination, might lead to remarkable improvement in the available therapies for several solid tumors poorly responsive or resistant to targeted therapies and/or chemotherapies.

## Author Contributions

All authors listed have made a substantial, direct and intellectual contribution to the work, and approved it for publication.

## Conflict of Interest

The authors declare that the research was conducted in the absence of any commercial or financial relationships that could be construed as a potential conflict of interest.
